# Cocoa Powder Modulates HIF-1α Stability and Inhibits Ocular Angiogenic and Degenerative Pathology

**DOI:** 10.3390/nu18071150

**Published:** 2026-04-03

**Authors:** Su Jung Hwang, InWha Park, Yeo Jin Sa, Kyu Ha Lee, Chung Sub Kim, Hyo-Jong Lee

**Affiliations:** 1 School of Pharmacy, Sungkyunkwan University, Suwon 16419, Republic of Korea; sama3575@skku.edu (S.J.H.); inwha129@skku.edu (I.P.); chungsub.kim@skku.edu (C.S.K.); 2 Nexpharm Korea Co., Ltd., Cheongju-si 16841, Republic of Korea; jin860513@nexpharm.co.kr (Y.J.S.); khalee79@nexpharm.co.kr (K.H.L.); 3 Department of Biopharmaceutical Convergence, Sungkyunkwan University, Suwon 16419, Republic of Korea

**Keywords:** cocoa powder, dietary polyphenols, vascular inflammation, endothelial regeneration, HIF-1α signaling, metabolic reprogramming, angiogenesis, tissue remodeling, corneal neovascularization, retinal degeneration

## Abstract

**Background/Objectives**: Vascular inflammation and impaired endothelial regeneration contribute to chronic degenerative disorders, including ocular neovascularization and retinal degeneration. Nutritional bioactives that modulate molecular pathways governing angiogenesis and tissue remodeling represent promising adjunct strategies for vascular health. This study investigated whether cocoa powder (CP) regulates hypoxia-driven molecular signaling and attenuates vascular inflammation and degeneration. **Methods**: The vascular-modulatory effects of CP were examined in human umbilical vein endothelial cells (HUVECs) and in murine models of alkali-induced corneal neovascularization and *N*-methyl-*N*-nitrosourea (MNU)-induced retinal degeneration. Hypoxia-inducible factor-1α (HIF-1α) signaling and downstream angiogenic targets were assessed by Western blotting and quantitative PCR. Endothelial migration, tube formation, and transwell assays were performed to evaluate angiogenic responses. In vivo, oral CP (50 or 200 mg/kg) was administered, and vascular growth, inflammatory and remodeling markers, and retinal structural integrity were analyzed by histology, immunofluorescence, and protein expression. **Results**: At non-cytotoxic concentrations (0.1–1.0 μg/mL), CP suppressed hypoxia-induced HIF-1α protein stabilization without altering HIF-1α mRNA levels and reduced expression of VEGFA, EPO, and GLUT1. CP significantly inhibited VEGF-A-induced endothelial migration, network formation, and chemotactic invasion. In alkali-injured corneas, CP reduced the neovascularized area and downregulated VEGF, MMP2, MMP9, α-smooth muscle actin, and Ninj1, indicating attenuation of vascular inflammation and fibrotic remodeling. In the MNU model, CP preserved outer nuclear layer thickness, reduced glial activation (GFAP), maintained rhodopsin expression, and decreased MMP9 induction. **Conclusions**: CP functions as a nutritional modulator of hypoxia-responsive and inflammatory pathways, suppressing pathological angiogenesis while supporting structural preservation in degenerative vascular conditions. These findings highlight the translational potential of dietary polyphenol-rich interventions in regulating vascular inflammation and regeneration.

## 1. Introduction

Pathological angiogenesis is a central feature of several vision-threatening ocular disorders, including corneal neovascularization (CNV) and age-related macular degeneration (AMD) [1]. With the aging population, the global prevalence of AMD continues to increase, and vision loss associated with choroidal neovascularization or progressive photoreceptor degeneration represents a substantial clinical and socioeconomic burden [2]. Intravitreal anti-vascular endothelial growth factor (VEGF) agents, such as ranibizumab, aflibercept, bevacizumab, and pegaptanib, have significantly improved outcomes in neovascular disease. However, these therapies require repeated intraocular injections at monthly or bimonthly intervals, imposing logistical and economic burdens on patients and healthcare systems. In addition, therapeutic responses vary considerably and some patients exhibit incomplete resolution of exudation or develop fibrovascular remodeling despite continued treatment [3]. Importantly, no current therapy fully prevents long-term disease progression or completely restores retinal integrity [4]. These limitations underscore the need for complementary strategies that are safe, accessible, and suitable for long-term use. From a food material perspective, dietary components and functional ingredients with multi-target biological activities may offer practical adjunctive value, particularly for chronic ocular conditions characterized by sustained angiogenic and degenerative processes [5].

Hypoxia and metabolic stress orchestrate many cellular programs that initiate and sustain vascular and neuroretinal pathology [6,7]. Hypoxia-inducible factor-1 (HIF-1) is a master regulator of oxygen homeostasis and its stabilization promotes dimerization with HIF-1β and binding to hypoxia-response elements (HREs), thereby inducing genes involved in angiogenesis and metabolic adaptation, including vascular endothelial growth factor (VEGF), erythropoietin (EPO), and glucose transporter-1 (GLUT-1) [8,9]. In ocular tissues, dysregulated HIF-1/VEGF signaling promotes endothelial proliferation, vascular permeability, and inflammatory remodeling [10,11]. These molecular changes are accompanied by tissue-level processes that facilitate pathological neovascularization and fibrosis. Matrix metalloproteinases (MMP2 and MMP9) degrade extracellular matrix components such as collagen IV and laminin, weakening the basement membrane and enabling endothelial migration during neovascular sprouting [12,13,14]. Myofibroblast activation, which is characterized by increased α-smooth muscle actin (α-SMA) expression, contributes to tissue contraction and fibrovascular membrane formation in proliferative vitreoretinopathy and neovascular AMD [15,16]. Additionally, reactive Müller glia, identified by upregulated glial fibrillary acidic protein (GFAP) expression, extend their processes beyond the endfeet into retinal layers under stress, contributing to gliosis and chronic inflammation [17,18]. Because angiogenic, inflammatory, and fibrotic responses develop in concert, dietary functional materials capable of modulating the HIF-1/VEGF axis together with downstream remodeling pathways may provide complementary benefit in chronic ocular disorders [12,19,20].

Cocoa powder (CP) is a widely consumed dietary ingredient containing abundant flavanol and procyanidin polyphenols [21,22]. In cardiovascular and metabolic settings, cocoa-based preparations have been associated with antioxidant effects, attenuation of low-grade inflammation, and improvement in endothelial function and microvascular reactivity [22,23,24,25]. These biological activities are mechanistically relevant to ocular disorders, in which oxidative stress, inflammatory signaling, and vascular dysfunction contribute to disease progression. Despite this evidence, the effects of CP on ocular angiogenesis and retinal degeneration have not been well characterized. In particular, it remains unclear whether CP modulates hypoxia-driven signaling pathways, including HIF-1α stabilization, suppresses VEGF-dependent endothelial activation, or attenuates glial reactivity and fibrotic marker expression in experimental models of AMD and corneal neovascularization.

In the present study, we evaluated CP as a food-derived material for ocular health using mechanism-oriented and quantitatively defined endpoints. At the cellular level, we examined whether CP attenuates hypoxia-induced stabilization of HIF-1α and reduces expression of its downstream targets in a hypoxic model and whether CP suppresses VEGF-A-induced angiogenic responses in human umbilical vein endothelial cells, as assessed by wound migration, tube formation, and transwell migration assays with quantitative image analysis. At the tissue level, in vivo relevance was investigated using (i) an alkali-induced corneal injury model that induces corneal neovascularization (CNV) with associated matrix remodeling and inflammatory responses and (ii) an N-methyl-N-nitrosourea (MNU)-induced dry AMD model characterized by photoreceptor degeneration, Müller gliosis, and thinning of the outer nuclear layer (ONL) [26]. In the MNU model, we quantified molecular and structural markers representing distinct aspects of retinal pathology, including VEGF (hypoxia-associated signaling), MMP2/MMP9 (extracellular matrix remodeling), α-SMA (fibrotic activation), GFAP (Müller glial reactivity), rhodopsin (photoreceptor integrity), and ONL thickness (structural preservation) [27]. By integrating molecular pathway analysis with histological endpoints, we aimed to determine whether CP modulates the HIF-1/VEGF axis and associated inflammatory–fibrotic cascades, thereby reducing pathological angiogenesis and retinal degeneration. We hypothesized that CP attenuates hypoxia-dependent signaling and downstream remodeling processes, supporting its potential complementary role in ocular neovascular and degenerative conditions.

## 2. Materials and Methods

### 2.1. Materials and Reagents

Cocoa powder (100% pure) was purchased from Dutch Cocoa BV (Amsterdam, The Netherlands), which supplies Dutch processed cocoa. For cell-based experiments, CP was dissolved in sterile distilled water and filtered through a 0.22 μm syringe filter prior to use. TRIzol reagent was obtained from Invitrogen (Carlsbad, CA, USA). Anti-HIF-1α antibody was purchased from BD Biosciences (San Jose, CA, USA). Antibodies against VEGF, MMP2, MMP9, α-SMA, and GAPDH were obtained from Santa Cruz Biotechnology (Santa Cruz, CA, USA). Antibodies against GFAP and rhodopsin and HRP-conjugated secondary antibodies were purchased from Cell Signaling Technology (Danvers, MA, USA). Ninj1 antibody was kindly provided by Dr. Kyu-Won Kim (Seoul National University, Republic of Korea). Dimethyl sulfoxide (DMSO), MTT, N-methyl-N-nitrosourea (MNU), sodium hydroxide (NaOH), and other standard reagents were purchased from Sigma-Aldrich (St. Louis, MO, USA). Growth factor-reduced Matrigel was obtained from Corning (Corning, NY, USA). Theobromine and caffeine (>98% purity) were purchased from TCI (Tokyo, Japan).

### 2.2. Chemical Characterization of Cocoa Powder

For chemical profiling, CP(150 mg) was extracted with 3 mL of 30% ethanol (EtOH) at room temperature for 24 h. To assess consistency with the preparation used in the in vitro assays, an aqueous extract was also prepared using the same amount of CP in distilled water under identical extraction conditions. The solvent was removed under reduced pressure at 35 °C, yielding 9.95 mg of ethanol extract residue. The residue was reconstituted in 30% methanol (MeOH) to a final concentration of 1 mg/mL. Authentic standards of theobromine and caffeine were dissolved in MeOH at 0.1 mg/mL. All solutions were filtered through a 0.45 μm cellulose acetate syringe filter (SPL Life Sciences, Pocheon, Republic of Korea) prior to analysis.

Chemical constituents of the ethanol extract were analyzed by high-performance liquid chromatography–mass spectrometry (LC–MS) using an Agilent 1260 HPLC system coupled to a 6130 single-quadrupole mass selective detector equipped with an electrospray ionization (ESI) source (Agilent Technologies, Santa Clara, CA, USA). Separation was achieved on a Phenomenex Kinetex C18 column (250 × 4.6 mm, 5 μm). The mobile phase consisted of water (A) and acetonitrile (B), each containing 0.1% formic acid, delivered at a flow rate of 0.7 mL/min. A linear gradient from 10% to 100% B over 20 min was applied. The injection volume was 10 μL, and chromatographic detection was performed at 210 nm.

### 2.3. Cell Culture

Immortalized human umbilical vein endothelial cells (HUVECs; HUEhT-1, #JCRB1458) were cultured in endothelial growth medium-2 (EGM-2; Lonza, Basel, Switzerland) supplemented with 2% fetal bovine serum (FBS), growth supplements, 20 μg/mL G418, 100 U/mL penicillin, and 100 μg/mL streptomycin. Cells were maintained at 37 °C in a humidified atmosphere containing 5% CO_2_ and used between passages 3 and 6.

### 2.4. Cell Viability Assay

HUVECs were seeded in 96-well plates at a density of 4 × 10^3^ cells per well and treated with cocoa powder (CP; 0.01–10 μg/mL) for 24 h. After treatment, the culture medium was replaced with fresh medium containing 10 μL of MTT reagent (3-(4,5-dimethylthiazol-2-yl)-2,5-diphenyltetrazolium bromide; Promega, Madison, WI, USA), followed by incubation at 37 °C for 2 h. The resulting formazan crystals were dissolved in dimethyl sulfoxide (DMSO: Sigma-Aldrich, St. Louis, MO, USA; D8418), and absorbance was measured at 490 nm using a microplate reader (Agilent Technologies, Santa Clara, CA, USA). Cell viability was calculated as a percentage relative to untreated controls. All treatments were performed in triplicate wells, and each experiment was independently repeated at least three times.

### 2.5. In Vitro Angiogenesis Assays (Wound Healing, Tube Formation, and Transwell Migration)

HUVECs were incubated with CP and VEGF-A (50 ng/mL; Sigma-Aldrich, St. Louis, MO, USA; SRP3182) as indicated. For scratch wound migration, confluent monolayers in 6-well plates were scratched with a sterile 1000 µL tip, rinsed with PBS, and incubated in EGM-2 containing 1–2% FBS with VEGF-A ± CP; images were acquired at 0 and 24 h using an Eclipse Ts2 microscope (Nikon, Tokyo, Japan), and the number of migrated cells into the wound area was quantified in ImageJ (NIH) relative to the VEGF-A group. For tube formation, 48-well plates were coated with growth factor-reduced Matrigel (Corning, Corning, NY, USA; 354234; 50 µL/well, 30 min, 37 °C), HUVECs (1 × 10^4^ cells/well) were seeded in serum-reduced medium with VEGF-A ± CP and incubated for 6 h, and branch points were quantified. For transwell migration, cells (5 × 10^4^) in serum-free medium ± CP were placed in 8 µm pore inserts (Corning, Corning, NY, USA; 3422) with VEGF-A (50 ng/mL) in the lower chamber; after 12 h, migrated cells were fixed and crystal-violet-stained, and cell numbers were counted. Data are expressed as mean ± SD from ≥3 independent experiments and analyzed by one-way ANOVA with appropriate post hoc tests (*p* < 0.05 considered significant).

### 2.6. MNU-Induced Dry AMD Mouse Model

Male C57BL/6 mice (7 weeks old) were obtained from Orient Bio Inc. (Seongnam, Republic of Korea). Animals were housed at 23 ± 2 °C under a 12 h light/12 h dark cycle with free access to standard laboratory chow and water. All experimental procedures were conducted in accordance with the Institutional Animal Care and Use Committee (IACUC) guidelines of Sungkyunkwan University and were approved under protocol number SKKUIACUC2022-07-04-1 (approved on 2 August 2022). A total of 36 mice were used in this experiment. To evaluate the protective effect of CP, mice were randomly divided into four experimental groups (n = 9 per group): (1) normal control, (2) MNU-injured control, (3) MNU + CP 50 mg/kg, and (4) MNU + CP 100 mg/kg. Mice were anesthetized using isoflurane inhalation during all procedures. In the injured groups (groups 2–4), retinal degeneration was induced by a single intraperitoneal injection of N-methyl-N-nitrosourea (MNU; MedChemExpress, Monmouth Junction, NJ, USA; HY-34758; 60 mg/kg), freshly dissolved in 0.05% acetic acid. CP was suspended in 0.5% sodium carboxymethyl cellulose (CMC; Sigma-Aldrich, St. Louis, MO, USA; 419273) and administered by oral gavage at doses of 50 or 100 mg/kg once on the day prior to MNU injection and subsequently once daily for 7 consecutive days. Control animals received an equivalent volume of 0.5% CMC vehicle. At the end of the treatment period, mice were euthanized under anesthesia and eyes were harvested for histological analysis (hematoxylin and eosin staining), immunofluorescence staining (GFAP and rhodopsin), and Western blot analysis (GFAP, rhodopsin, and MMP9).

### 2.7. Alkali-Induced Corneal Injury Model

Seven-week-old female ICR mice were anesthetized using isoflurane inhalation prior to injury induction. A total of 30 mice were used in this experiment. Mice were randomly assigned to each experimental group (n = 7 for control and CP-treated groups, n = 9 for the alkali-injured group). A 1–2 mm diameter filter paper disc soaked in NaOH (Sigma-Aldrich, St. Louis, MO, USA; 221465) solution was applied to the central cornea of the right eye for 30 s, followed by immediate irrigation with 10 mL of sterile saline. CP was suspended in 0.5% sodium carboxymethyl cellulose (CMC) and administered orally (50 or 100 mg/kg) once on the day prior to injury and once daily for 7 consecutive days thereafter. Control and alkali-injured groups received an equivalent volume of vehicle (0.5% CMC). Corneal neovascularization (CNV) was assessed on day 7 post-injury using a slit-lamp microscope. CNV severity was graded on a 0–4 scale as follows: 0, no vessels extending beyond the limbus; 1, vessels extending ≤1 mm from the limbus; 2, vessels extending ≤2 mm from the limbus; 3, vessels extending toward the central cornea (≥4 mm from the limbus); and 4, vessels extending within ≤2 mm of the corneal center. All procedures were conducted in accordance with the Institutional Animal Care and Use Committee (IACUC) guidelines of Sungkyunkwan University and were approved under protocol number SKKUIACUC2022-06-51-1 (approved on 28 July 2022). At the end of the experiment, mice were euthanized under anesthesia.

### 2.8. Protein Extraction and Western Blot Analysis

HUVECs pretreated with cocoa powder (CP; 0.1–1.0 μg/mL) and ocular tissues obtained from mice orally administered CP (50 or 200 mg/kg) were processed for protein analysis. Cells were washed with ice-cold phosphate-buffered saline (PBS) and lysed on ice in RIPA buffer supplemented with protease and phosphatase inhibitor cocktails GenDEPOT, Katy, TX, USA; Cat. No. P3100-003. Whole eye tissues were homogenized in the same lysis buffer and centrifuged at 12,000–14,000× *g* for 10 min at 4 °C to remove debris. Protein concentrations were determined using a bicinchoninic acid (BCA) assay (GenDEPOT, Katy, TX, USA; Cat. No. P8101-050). Equal amounts of protein (30 μg per sample) were separated by 10–12% SDS–polyacrylamide gel electrophoresis (SDS–PAGE) and transferred onto nitrocellulose membranes. Membranes were blocked with 5% nonfat dry milk in Tris-buffered saline containing 0.1% Tween-20 (TBST) for 1 h at room temperature, followed by incubation with primary antibodies overnight at 4 °C. After washing, membranes were incubated with horseradish peroxidase (HRP)-conjugated secondary antibodies for 1 h at room temperature. Immunoreactive bands were visualized using enhanced chemiluminescence (ECL) substrate (iNtRON Biotechnology, Seongnam, Republic of Korea) and detected with a FUSION-SL4 chemiluminescence imaging system (Vilber Lourmat, Marne-la-Vallée, France). Band intensities were quantified by densitometric analysis and normalized to the respective loading controls. Relative protein expression levels were expressed as fold change compared with the control group.

### 2.9. Measurement of TNF-α Levels in Alkali-Burned Mouse Eyes

To evaluate the inflammatory response in the alkali-burned eye model, the concentration of TNF-α in eye tissues was measured using a commercial BD OptEIA™ Mouse TNF-α ELISA Set (Cat. No. 555268; BD Biosciences, San Diego, CA, USA) according to the manufacturer’s instructions. Briefly, eye tissues were homogenized in a lysis buffer containing protease inhibitors. After centrifugation at 12,000 rpm for 15 min at 4 °C, the supernatants were collected, and the total protein concentration was determined using a BCA protein assay kit. Subsequently, 100 μg of the prepared tissue lysates (or standards) were added to each well of a 96-well plate previously coated with a capture antibody and blocked with 10% FBS in PBS. After incubation for 2 h, a working detector (biotinylated detection antibody and streptavidin–horseradish peroxidase conjugate) was added. The reaction was developed using a TMB substrate solution and stopped with a stop solution (H_2_SO_4_). The absorbance was measured at 450 nm using a microplate reader. The final concentration of TNF-α was calculated using a standard curve and expressed relative to the total protein content.

### 2.10. RNA Isolation and Quantitative Real-Time PCR

Total RNA was extracted from HUVECs and mouse ocular tissues using TRIzol reagent (Invitrogen, Carlsbad, CA, USA) according to the manufacturer’s protocol. RNA purity and quantity were assessed by spectrophotometry (A260/A280), and 5 µg of RNA was reverse-transcribed to cDNA using a standard reverse transcription procedure. Quantitative PCR was performed on a Rotor-Gene Q cycler (Qiagen, Germantown, MD, USA) using the Rotor-Gene SYBR Green RT-PCR Kit (Qiagen). Reactions (20 µL) contained 1× SYBR Green master mix, gene-specific primers (0.2–0.4 µM each), and a cDNA template. The cycling program was: 95 °C for 5 min and 40 cycles of 95 °C for 10 s and 60 °C for 20–30 s. A melt-curve analysis (65–95 °C) confirmed single amplicons. No-template controls were included in every run. Primer sequences and amplicon sizes are summarized in Table S1. Expression was normalized to 18S rRNA (human or mouse), and relative mRNA levels were calculated by the 2^−ΔΔCt^ method. All samples were analyzed in technical triplicates, and data represent at least three independent experiments.

### 2.11. Immunofluorescence Microscopy and Quantification

OCT-embedded retinal sections were permeabilized with 0.1% Triton X-100 in phosphate-buffered saline (PBS) for 10 min and blocked with 5% bovine serum albumin (BSA) in PBS for 1 h at room temperature. Sections were then incubated overnight at 4 °C with primary antibodies against glial fibrillary acidic protein (GFAP) and rhodopsin (Cell Signaling Technology, Danvers, MA, USA). After washing with PBS, sections were incubated with appropriate fluorescent secondary antibodies (Cell Signaling Technology) for 1 h at room temperature and counterstained with 4′,6-diamidino-2-phenylindole (DAPI; Sigma-Aldrich, St. Louis, MO, USA). Slides were mounted using antifade mounting medium. Images were acquired using an Eclipse Ts2 fluorescence microscope (Nikon, Tokyo, Japan) under identical exposure and gain settings for all groups. Scale bars are indicated in the corresponding figures. Fluorescence intensity was quantified using ImageJ software (version 1.54g; National Institutes of Health, Bethesda, MD, USA). Mean fluorescence values were normalized to the vehicle control group (defined as 100%) and expressed as mean ± standard deviation (SD) from at least three independent experiments.

### 2.12. Statistical Analysis

Data are reported as mean ± standard deviation (SD) from triplicate experiments and, where indicated, were normalized to the corresponding control and are expressed as relative values. Statistical testing was performed in GraphPad Prism v6 (GraphPad Software, San Diego, CA, USA) using either an unpaired two-tailed Student’s *t* test or one-way ANOVA followed by Tukey’s multiple-comparisons test. Differences were considered significant at *p* < 0.05.

## 3. Results

### 3.1. Cocoa Powder Composition Was Characterized by LC–MS Analysis

**Figure 1:**
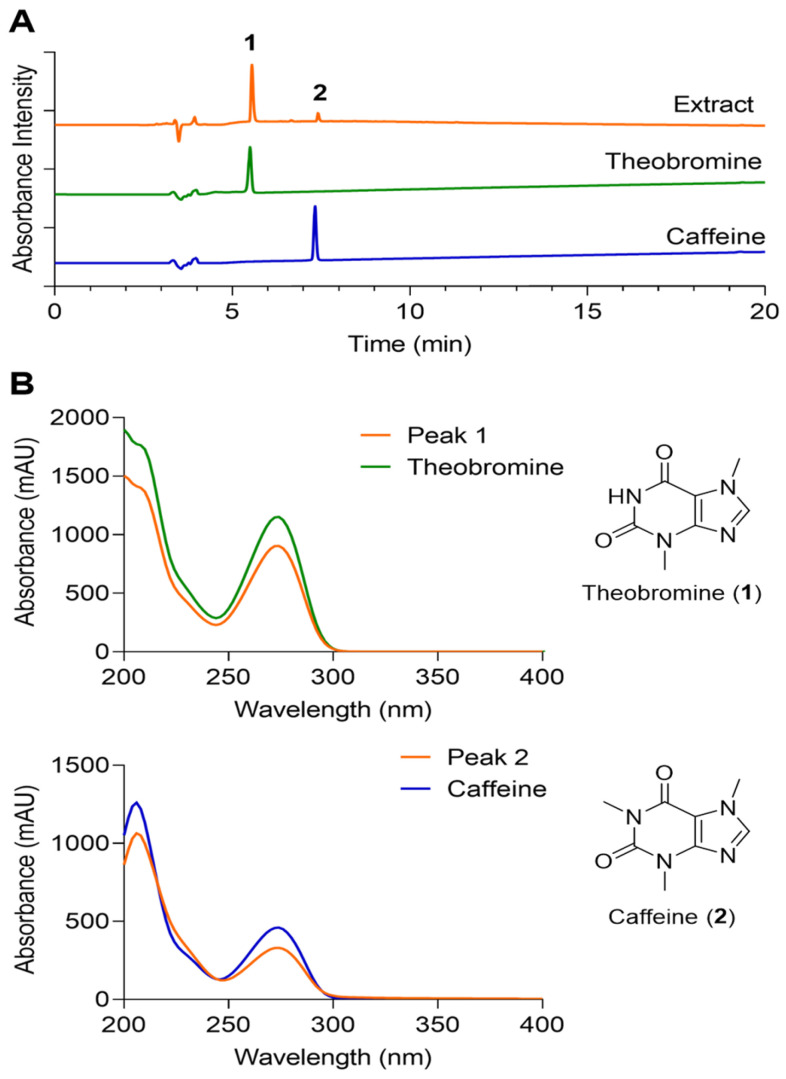
LC–MS analysis of cocoa extract and standards. (**A**) UV chromatograms at 210 nm of the cocoa extract and standards. (**B**) UV spectra of peaks 1 and 2 and corresponding standards.

For chemical characterization, both the 30% ethanolic extract and the aqueous preparation of CP were analyzed by LC–MS. In both preparations, two predominant peaks corresponding to theobromine and caffeine were identified by comparison with authentic standards (Figure 1). These results indicate that the major detectable constituents were consistent between the analytically characterized extract and the preparation used for cell-based assays. Notably, the CP used in this study was Dutch-processed CP, which is treated with alkali. This process has been reported to markedly reduce flavanols due to oxidation and polymerization [28,29]. Accordingly, these alkaloids represent characteristic constituents of CP and support the chemical identity of the material used in this study (Figure 1 and Table 1).

### 3.2. Cocoa Powder Attenuated Hypoxia-Induced Stabilization of HIF-1α and Downstream Gene Expression Without Cytotoxicity

**Figure 2:**
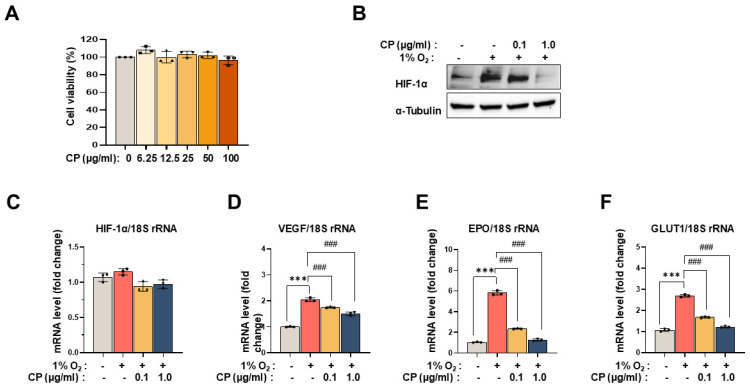
Effect of cocoa powder (CP) on hypoxia-inducible factor-1α (HIF-1α) stability and downstream target gene expression in human umbilical vein endothelial cells (HUVECs). (**A**) HUVECs were treated with various concentrations of CP (0–100 μg/mL) for 24 h, and cell viability was determined by MTT assay. (**B**) Cells were pretreated with CP (0.1 or 1.0 μg/mL) for 24 h and subsequently exposed to hypoxia for 6 h. HIF-1α protein levels were determined by Western blotting, and α-tubulin was used as a loading control. (**C**) mRNA expression of HIF-1α was analyzed by real-time PCR. (**D**–**F**) mRNA expression levels of HIF-1α target genes including vascular endothelial growth factor (VEGF), erythropoietin (EPO), and glucose transporter 1 (GLUT1). Values are expressed as mean ± SD (n = 3). *** *p* < 0.001 vs. normoxia; ### *p* < 0.001 vs. hypoxia.

To determine a non-cytotoxic concentration range, HUVECs were treated with CP (0–100 μg/mL) for 24 h, and cell viability was assessed using the MTT assay. CP did not significantly affect cell viability at concentrations up to 1.0 μg/mL, whereas higher concentrations resulted in moderate cytotoxicity (Figure 2A). Based on these findings, 0.1 and 1.0 μg/mL were selected for subsequent experiments. Under hypoxic conditions, HIF-1α protein levels were markedly increased compared with normoxia. Pretreatment with CP (0.1 or 1.0 μg/mL) for 24 h prior to hypoxia exposure (6 h) significantly reduced hypoxia-induced accumulation of HIF-1α protein (Figure 2B). In contrast, CP did not alter HIF-1α mRNA expression under hypoxic conditions (Figure 2C). Consistent with the reduction in HIF-1α protein, CP significantly decreased hypoxia-induced mRNA expression of downstream target genes, including VEGF, EPO, and GLUT1 (Figure 2D–F). These results demonstrate that CP attenuates hypoxia-induced HIF-1α protein stabilization and downstream gene expression at non-cytotoxic concentrations.

### 3.3. Cocoa Powder Suppressed VEGF-A-Induced Angiogenic Activities in HUVECs

**Figure 3:**
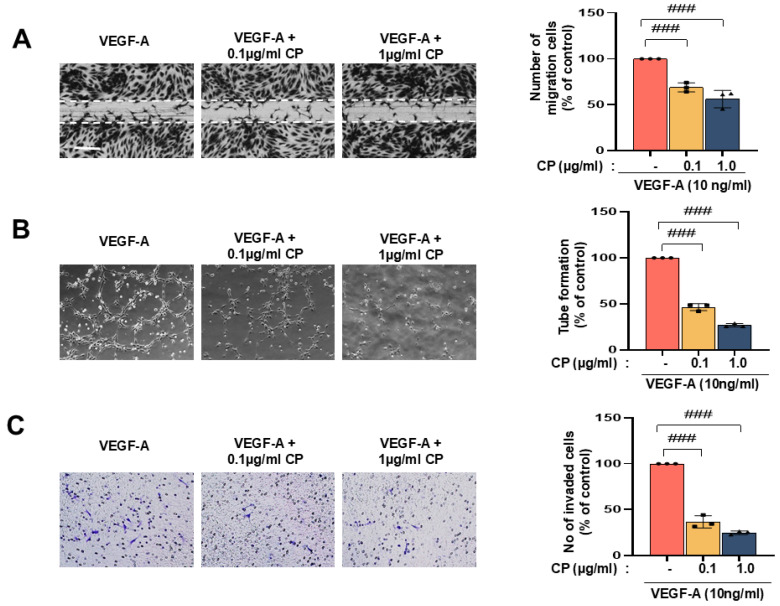
Effect of CP on VEGF-A-induced angiogenesis in HUVECs. (**A**) Scratch wound healing assay showing the inhibitory effect of CP on VEGF-A-stimulated endothelial cell migration. HUVECs were pretreated with CP (0.1 or 1.0 μg/mL) and subsequently exposed to VEGF-A (10 ng/mL) for 24 h. Representative images of wound closure are shown, and quantitative analysis demonstrates a dose-dependent suppression of VEGF-A-induced migration by CP. (**B**) Tube formation assay demonstrating that CP attenuates VEGF-A-induced capillary-like network formation. HUVECs were seeded on Matrigel-coated plates and treated with VEGF-A in the absence or presence of CP (0.1 or 1.0 μg/mL) for 6 h. Representative images show tubular structures, and quantitative analysis revealed a marked reduction in total tube length upon CP treatment. (**C**) Transwell migration assay showing the suppressive effect of CP on VEGF-A-induced chemotactic migration. HUVECs were seeded in the upper chamber with CP, while VEGF-A (10 ng/mL) was added to the lower chamber. After 12 h, migrated cells were stained and quantified. CP significantly decreased the number of VEGF-A-induced migrated cells in a concentration-dependent manner. Values are expressed as mean ± SD (n = 3). ### *p* < 0.001 vs. VEGF-A-treated group.

To evaluate the effect of CP on VEGF-A-induced angiogenic responses, functional assays were performed in HUVECs. In the scratch wound migration assay, VEGF-A stimulation resulted in approximately 80% wound closure at 24 h. Pretreatment with CP significantly reduced VEGF-A-induced migration in a concentration-dependent manner, with wound closure decreasing to approximately 55% at 0.1 μg/mL and 40% at 1.0 μg/mL (Figure 3A). In the tube formation assay, VEGF-A markedly increased capillary-like network formation, which was defined as 100% relative to the VEGF-A-treated group. CP treatment significantly reduced total tube length to approximately 70% at 0.1 μg/mL and 45% at 1.0 μg/mL (Figure 3B). Similarly, in the transwell migration assay, VEGF-A promoted endothelial chemotactic migration, whereas CP significantly decreased the number of migrated cells by approximately 35% at 0.1 μg/mL and 55% at 1.0 μg/mL compared with VEGF-A alone (Figure 3C).

These data indicate that CP attenuates VEGF-A-induced endothelial migration and tube formation under the experimental conditions tested.

### 3.4. Cocoa Powder Attenuated Alkali-Induced Corneal Neovascularization

**Figure 4:**
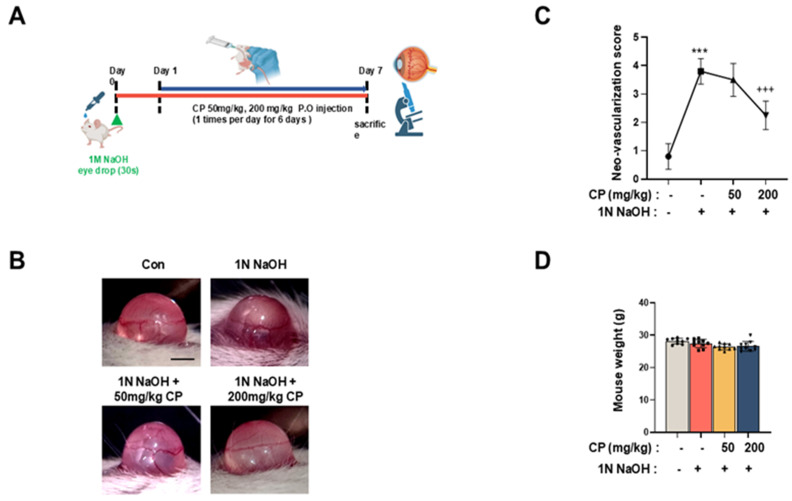
Effect of CP on alkali-induced corneal neovascularization in mice. (**A**) Schematic diagram of the experimental procedure. Corneal injury was induced by topical application of 1 N NaOH for 30 s, followed by extensive washing with PBS. Mice were orally administered vehicle or CP (50 or 200 mg/kg) once daily for 7 days. (**B**) Representative images of mouse corneas at day 7 after alkali burn, showing neovascularization in vehicle-treated and CP-treated groups. Scale bar = 0.5 mm. (**C**) Quantification of neovascularized area expressed as percentage of total corneal surface. (**D**) Body weight of mice during the experimental period. Values are expressed as mean ± SD (n = 7 for control and CP-treated groups, n = 9 for the alkali-injured group). *** *p* < 0.001 vs. normal control; +++ *p* < 0.001 vs. alkali-injured control.

The anti-angiogenic effect of CP was evaluated using a murine corneal alkali burn model (Figure 4A). At day 7 post-injury, extensive corneal neovascularization was observed in the alkali-injured group, with newly formed vessels covering approximately 65 ± 5% of the corneal surface. Oral administration of CP significantly reduced the neovascularized area in a dose-dependent manner. Treatment with CP at 50 mg/kg decreased vascular coverage to 38 ± 4%, whereas 200 mg/kg further reduced the neovascularized area to 22 ± 3% (Figure 4B,C). Body weight was monitored throughout the experimental period. No significant differences in body weight were observed between CP-treated and vehicle-treated groups (Figure 4D).

### 3.5. Cocoa Powder Downregulated Angiogenesis-, Inflammation-, and Fibrosis-Related Markers in Alkali-Injured Corneal Tissue

**Figure 5:**
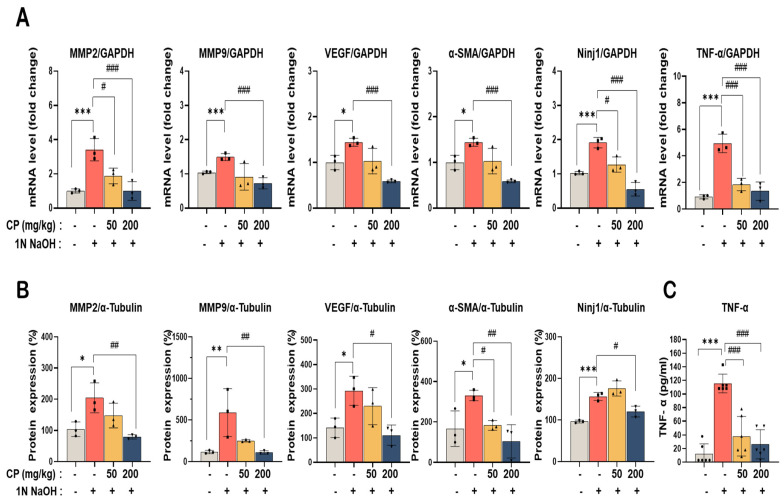
Effect of cocoa powder (CP) on angiogenesis-related biomarkers in corneal tissue after alkali injury. (**A**) mRNA expression levels of Vegfa, Tnf, Ninj1, Mmp2, Mmp9, and Atra2 were analyzed by real-time PCR using corneal tissues from mice subjected to alkali burn (1 N NaOH) and subsequently treated with vehicle or CP (50 or 200 mg/kg, oral administration, once daily for 7 days). (**B**) Protein expression of MMP2, MMP9, VEGF, α-SMA, and Ninj1 was determined by Western blotting. Tubulin was used as a loading control. (**C**) TNF-α protein concentration in the corneal tissue lysates was measured by ELISA. Densitometric quantification of protein expression normalized to α-tubulin. Values are expressed as mean ± SD (n = 3 per group). * *p* < 0.05, ** *p* < 0.01, *** *p* < 0.001 vs. normal control; # *p* < 0.05, ## *p* < 0.01, ### *p* < 0.001 vs. alkali-injured control.

To assess molecular changes in alkali-injured corneal tissue, mRNA expression levels of angiogenesis- and inflammation-related genes were analyzed by quantitative real-time PCR. Alkali injury significantly increased the transcript levels of Vegfa, Tnf, Ninj1, Mmp2, Mmp9, and Atra2 compared with the normal control group. Oral administration of CP significantly reduced these elevations in a dose-dependent manner. At 50 mg/kg, CP decreased gene expression by approximately 35–45%, whereas 200 mg/kg reduced transcript levels by approximately 60% relative to the alkali-injured group (Figure 5A). At the protein level, Western blot analysis showed that alkali injury markedly increased expression of VEGF, MMP2, MMP9, α-SMA, and Ninj1. CP treatment reduced the expression of these proteins in a dose-dependent manner (Figure 5B). Densitometric analysis indicated that 200 mg/kg CP decreased MMP2 and MMP9 levels by approximately 50%, VEGF by more than 60%, and α-SMA and Ninj1 by more than 55% compared with the alkali-injured group (Figure 5B). Furthermore, the protein concentration of TNF-α was quantitatively measured by ELISA to further validate the anti-inflammatory effect of CP. As shown in Figure 5C, alkali injury induced a dramatic increase in TNF-α levels (approximately 115 pg/mL), which was significantly suppressed by CP treatment at doses of 50 and 200 mg/kg (approximately 67% and 78% reduction, respectively).These data demonstrate that CP reduced the expression of multiple markers associated with angiogenesis, inflammation, and fibrotic remodeling in alkali-injured corneal tissue.

### 3.6. Cocoa Powder Attenuated MNU-Induced Retinal Degeneration

**Figure 6:**
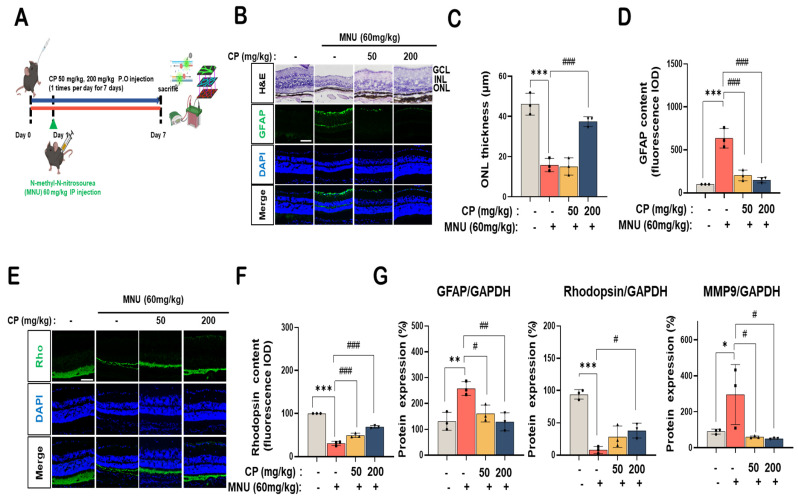
Effect of cocoa powder (CP) on retinal degeneration in MNU-induced dry AMD mouse model. (**A**) Experimental design of the study (n = 9 animals per group). Retinal degeneration was induced by intraperitoneal injection of N-methyl-N-nitrosourea (MNU, 60 mg/kg), and mice were treated with vehicle or CP (50 or 200 mg/kg, oral administration). (**B**) Retinal histology with H&E staining and immunofluorescence analysis of GFAP (glial fibrillary acidic protein, green), DAPI (blue), and merged images. GCL: ganglion cell layer; INL: inner nuclear layer; ONL: outer nuclear layer. (**C**) Quantification of ONL thickness. * *p* < 0.05, ** *p* < 0.01, *** *p* < 0.001 vs. vehicle control; # *p* < 0.05, ## *p* < 0.01, ### *p* < 0.001 vs. MNU-treated group. (**D**) Quantification of GFAP immunofluorescence intensity expressed as relative values compared with vehicle control (100% ± SD). * *p* < 0.05, ** *p* < 0.01, *** *p* < 0.001 vs. vehicle control; # *p* < 0.05, ## *p* < 0.01, ### *p* < 0.001 vs. MNU-treated group. IOD: integrated optical density. (**E**) Immunofluorescence staining of rhodopsin (Rho, green), DAPI (blue), and merged images. (**F**) Quantification of rhodopsin immunofluorescence intensity expressed as relative values compared with vehicle control (100% ± SD). * *p* < 0.05, ** *p* < 0.01, *** *p* < 0.001 vs. vehicle control; # *p* < 0.05, ## *p* < 0.01, ### *p* < 0.001 vs. MNU-treated group. (**G**) Western blot analysis of GFAP, rhodopsin, and MMP9 protein expression in whole eye extracts. GAPDH was used as a loading control. Densitometric quantification of Western blot results normalized to GAPDH and expressed relative to vehicle control (100% ± SD). * *p* < 0.05, ** *p* < 0.01, *** *p* < 0.001 vs. vehicle control; # *p* < 0.05, ## *p* < 0.01, ### *p* < 0.001 vs. MNU-treated group.

The protective effect of CP was evaluated in an MNU-induced dry AMD mouse model (Figure 6A). Hematoxylin and eosin staining showed that vehicle-treated retinas exhibited well-organized ganglion cell layer (GCL), inner nuclear layer (INL), and outer nuclear layer (ONL) structures (Figure 6B). In contrast, MNU administration resulted in marked retinal degeneration, characterized by thinning of the ONL and disruption of photoreceptor nuclei. CP treatment preserved retinal laminar organization and increased ONL thickness compared with the MNU group (Figure 6B). Quantitative analysis demonstrated that ONL thickness was reduced to approximately 40% of control following MNU injection, whereas CP treatment restored ONL thickness to approximately 65% and 80% at 50 and 200 mg/kg, respectively (Figure 6C). Immunofluorescence analysis revealed a marked increase in glial fibrillary acidic protein (GFAP) expression in MNU-treated retinas, with signal distribution extending across retinal layers (Figure 6B). CP treatment reduced GFAP fluorescence intensity in a dose-dependent manner. Quantification showed that GFAP levels were increased approximately three-fold after MNU exposure and were reduced by approximately 35% and 60% following treatment with 50 and 200 mg/kg CP, respectively (Figure 6D). Rhodopsin (Rho) immunostaining demonstrated a substantial reduction in photoreceptor outer segment signal following MNU administration (Figure 6E). CP treatment increased rhodopsin signal intensity compared with the MNU group. Quantitative analysis indicated that rhodopsin levels decreased to approximately 45% of control after MNU injection and were restored to approximately 70% and 85% with 50 and 200 mg/kg CP, respectively (Figure 6F). Western blot analysis showed that MNU exposure increased GFAP and MMP9 protein expression while decreasing rhodopsin levels (Figure 6G). CP treatment reduced GFAP and MMP9 expression and increased rhodopsin levels in a dose-dependent manner. Densitometric analysis indicated that GFAP and MMP9 were reduced by approximately 30–40% at 50 mg/kg and by more than 60% at 200 mg/kg, whereas rhodopsin expression increased from approximately 40% in the MNU group to 75% and 90% with 50 and 200 mg/kg CP, respectively.

## 4. Discussion

In the present study, cocoa powder (CP) was evaluated in mechanistically defined cellular and in vivo models of ocular angiogenesis and retinal degeneration. CP reduced hypoxia-induced stabilization of HIF-1α protein without altering HIF-1α mRNA levels and decreased expression of canonical downstream targets, including VEGFA, EPO, and GLUT1 (Figure 2). CP also suppressed VEGF-A-induced endothelial migration and tube formation in vitro (Figure 3). In vivo, oral administration of CP reduced alkali-induced corneal neovascularization and decreased expression of angiogenesis-, remodeling-, and injury-related markers in corneal tissue (Figure 4). In the MNU-induced retinal degeneration model, CP preserved outer nuclear layer thickness, reduced GFAP expression, maintained rhodopsin levels, and decreased MMP9 expression (Figure 5). Collectively, these findings indicate that CP modulates hypoxia-responsive signaling and associated angiogenic and remodeling pathways in ocular tissues.

The reduction in HIF-1α protein in the absence of changes in HIF-1α mRNA suggests regulation at a post-transcriptional level rather than transcriptional suppression (Figure 2B,C). One possible explanation is enhanced degradation of HIF-1α via prolyl-hydroxylation and von Hippel–Lindau (VHL)-mediated proteasomal pathways or attenuation of oxidative stabilization [9,30]. The concomitant decrease in VEGFA, EPO, and GLUT1 transcripts is consistent with attenuation of hypoxia-driven transcriptional programs linked to angiogenesis and metabolic adaptation [8]. Previous studies have shown that cocoa flavanols improve endothelial function and reduce oxidative stress in cardiovascular settings [31,32], supporting the possibility that similar redox-sensitive mechanisms may contribute to the effects observed in ocular cells. However, an important consideration in interpreting the present data is that the cocoa powder (CP) used in this study was Dutch-processed cocoa powder (CP), for which flavanol content is expected to be reduced during alkalization. Consistent with this, LC–MS analysis identified theobromine and caffeine as the major detectable constituents of the tested material (Figure 1). Accordingly, the biological effects observed here should not be attributed solely to flavanols, but rather to the processed cocoa matrix as a whole, potentially involving remaining polyphenolic constituents. This compositional distinction should be considered when comparing the present findings with studies using flavanol-rich cocoa extracts or purified flavanol preparations.

The inhibition of endothelial migration and tube formation by CP corresponds to functional endpoints of angiogenesis (Figure 3). Human intervention studies have reported improvements in flow-mediated dilation and reductions in endothelial activation markers following cocoa flavanol intake [33,34]. In experimental models of vascular inflammation, cocoa flavanols reduced expression of adhesion molecules and NF-κB signaling components [35], which parallels the decreased expression of matrix remodeling and inflammatory markers observed in alkali-injured corneas in the present study (Figure 4). Although direct mechanistic equivalence cannot be assumed, these observations are consistent with multi-pathway modulation of vascular responses.

In the alkali injury model, CP reduced neovascular area in a dose-dependent manner and lowered expression of Vegfa, Mmp2, Mmp9, Atra2, and Ninj1 transcripts, together with corresponding reductions in VEGF, MMP2/MMP9, α-SMA, and Ninj1 proteins (Figure 5). These changes are compatible with reduced extracellular matrix remodeling and fibrotic activation, processes that facilitate pathologic vessel ingrowth. Similar reductions in oxidative stress and inflammatory mediators have been reported in systemic models following cocoa consumption [34]. The decrease in Ninj1 expression may also reflect modulation of inflammatory cell adhesion pathways previously implicated in vascular injury responses [36].

In the MNU model, preservation of ONL thickness is indicative of photoreceptor structural maintenance. CP treatment increased ONL thickness relative to MNU alone and reduced GFAP expression while maintaining rhodopsin levels (Figure 6B–F). Western blot analysis confirmed reductions in GFAP and MMP9 and restoration of rhodopsin (Figure 6G). These findings extend the effects of CP beyond vascular modulation to retinal structural parameters associated with degeneration. Although clinical evidence remains limited, an ancillary analysis of the COSMOS trial suggested a potential early protective association between cocoa flavanol supplementation and age-related macular degeneration risk, albeit without sustained long-term benefit [24]. Such findings underscore both the potential and the limitations of cocoa-based interventions in chronic ocular disease.

Beyond ocular contexts, cocoa flavanols have been reported to influence endothelial and immune function through genomic and epigenomic modulation of barrier-related pathways [35]. Modulation of redox-sensitive transcription factors such as NF-κB and Nrf2, as well as PI3K/Akt/mTOR and nitric oxide signaling, may intersect with HIF-1 regulation and contribute to coordinated changes in VEGF, MMPs, and GFAP expression. Further studies assessing reactive oxygen species levels and HIF-1α hydroxylation status would help clarify whether CP primarily enhances proteasomal degradation or attenuates oxidative stabilization of HIF-1α.

From a translational perspective, compositional characterization and standardization of cocoa powder (CP) preparations remain important considerations. In the present study, oral CP at 50–200 mg/kg produced measurable tissue-level effects (Figure 3, Figure 4 and Figure 5). Human studies have reported endothelial and inflammatory benefits with cocoa flavanol intake in the range of 150–1000 mg/day [31,33,34] although direct dose equivalence cannot be inferred. Careful alignment of flavanol content, matrix composition, and exposure levels will be required to contextualize these findings within dietary frameworks.

In summary, CP attenuated hypoxia-mediated HIF-1/VEGF signaling, reduced endothelial activation and matrix remodeling markers, and preserved retinal structural parameters in experimental models. These findings are consistent with previously reported vascular and anti-inflammatory properties of cocoa flavanols [24,31,32,33,34,35,37] and support further investigation of standardized cocoa powder (CP) as a complementary dietary approach in ocular neovascular and degenerative conditions.

## 5. Conclusions

Cocoa powder (CP) suppressed hypoxia-driven HIF-1α signaling, inhibited VEGF-A-induced endothelial angiogenic responses, and reduced vascular and inflammatory remodeling markers in experimental ocular injury models. In addition, cocoa powder (CP) preserved retinal structure and photoreceptor-associated markers in MNU-induced retinal degeneration. These findings suggest that CP functions as a dietary modulator of pathological angiogenesis and degenerative remodeling and may provide complementary benefit in ocular neovascular and retinal degenerative conditions. Further studies are needed to clarify the specific bioactive components and mechanisms responsible for these effects and to evaluate their translational applicability.

## Figures and Tables

**Table 1:** Peak information of two major compounds in cocoa powder extract analyzed by LC-MS.

Peak	*t*_R_ (min)	Assignment	MW	MS (*m*/*z*)
1	5.55	theobromine	180	181.1 [M+H]^+^
2	7.41	caffeine	194	195.1 [M+H]^+^

## Data Availability

No new data were created or analyzed in this study. Data sharing is not applicable to this article.

## Supplementary Materials

The following supporting information can be downloaded at https://www.mdpi.com/article/10.3390/nu18071150/s1, Table S1: Primer sequences and expected product sizes used for qPCR analysis. Figure S1. Comparison of LC-MS chromatogram of 30% EtOH extract and the 100% aqueous extract.

## Abbreviations

The following abbreviations are used in this manuscript:

CP	Cocoa powder
HIF-1α	Hypoxia-inducible factor-1 alpha
VEGF	Vascular endothelial growth factor
CNV	Corneal neovascularization
AMD	Age-related macular degeneration
MNU	N-methyl-N-nitrosourea
ONL	Outer nuclear layer
GFAP	Glial fibrillary acidic protein
MMP	Matrix metalloproteinase
HUVEC	Human umbilical vein endothelial cell
EPO	Erythropoietin
GLUT1	Glucose transporter 1
α-SMA	Alpha-smooth muscle actin
