# Supplementary material for: Cocoa Powder Modulates HIF-1α Stability and Inhibits Ocular Angiogenic and Degenerative Pathology
Source: Nutrients. 2026 Apr 3;18(7):1150. doi: 10.3390/nu18071150 (PMC13075018; doi:10.3390/nu18071150)
Supplement: Supplementary file 1 [file nutrients-18-01150-s001.zip › nutrients-4217758-supplementary.pdf]

Supplementary Materials

Supplementary Figure S1. Comparison of LC-MS chromatogram of 30% EtOH extract and the 100% aqueous extract

<table>
<tr><td>Extraction solvent</td><td></td><td>LC-MS chromatogram</td></tr>
<tr><td rowspan="2">30% EtOH extract</td><td>195 nm</td><td>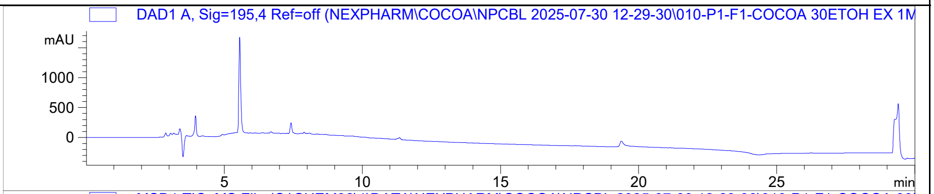
</td></tr>
<tr><td>TIC</td><td>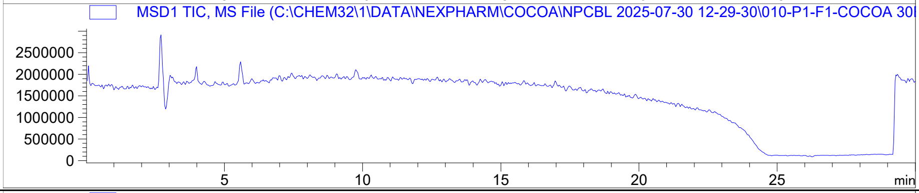
</td></tr>
<tr><td rowspan="2">100% aqueous extract</td><td>195 nm</td><td>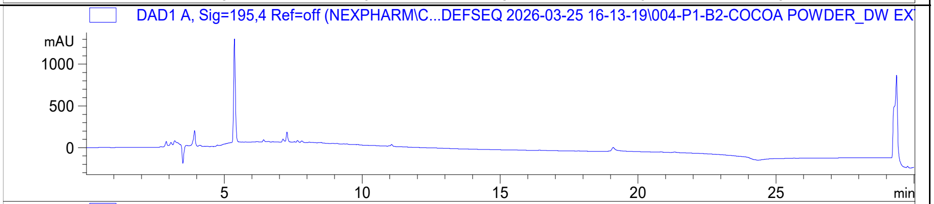
</td></tr>
<tr><td>TIC</td><td>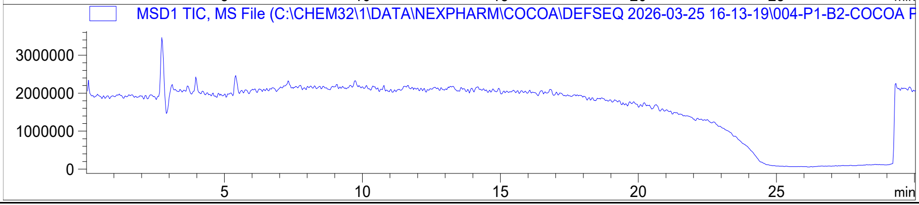
</td></tr>
</table>

Table S1. Primer sequences and expected product sizes used for qPCR analysis.

Gene	Species	Primer Sequence	Product size (bp)
HIF1A	Human	F: GAACGTCGAAAAGAAAAGTCTCG<br>R: CCTTATCAAGATGCGAACTCACA	124 bp
VEGF	Human	F: AGGGCAGAATCATCACGAAGT	75 bp

		R: AGGGTCTCGATTGGATGGCA	
EPO	Human	F: GGAGGCCGAGAATATCACGAC<br>R: CCCTGCCAGACTTCTACGG	136 bp
GLUT1	Human	F: CATCATCTTCATCCCGGC<br>R: CTCCTCGTTGCGGTTGAT	89 bp
18S rRNA	Human	F: GGACAGGATTGACAGATTGATA<br>R: CTCGTTCGTTATCGGAATTAA	106 bp
Vegf	Mouse	F: GCACATAGAGAGAATGAGCTTCC<br>R: CTCCGCTCTGAACAAGGCT	103 bp
Tnf-α	Mouse	F: GGTGCCTATGTCTCAGCCTCTT<br>R: GCCATAGAACTGATGAGAGGGAG	137 bp
Ninj1	Mouse	F: GCAGGGCAATGATTTCGCCTTCTT<br>R: ACTTGACCAGGAAGATGAGCAGCA	102 bp
Gapdh	Mouse	F: GAGCTGAACGGGAAGCTCACTGG<br>R: CCACCTTCTTGATGTCATCAT	116 bp
